# Variation in menopausal vasomotor symptoms outcomes in clinical trials: a systematic review

**DOI:** 10.1111/1471-0528.15990

**Published:** 2019-11-13

**Authors:** S Iliodromiti, W Wang, MA Lumsden, MS Hunter, R Bell, G Mishra, M Hickey

**Affiliations:** ^1^ Women’s Health Division Blizard Institute Queen Mary University London London UK; ^2^ School of Medicine University of Glasgow Glasgow UK; ^3^ Department of Obstetrics Shanghai First Maternity and Infant Hospital Tongji University School of Medicine Shanghai China; ^4^ Institute of Psychiatry, Psychology and Neuroscience Kings College London London UK; ^5^ Women’s Health Research Program School of Public Health and Preventive Medicine Monash University Melbourne Australia; ^6^ Faculty of Medicine School of Public Health University of Queensland Brisbane Australia; ^7^ Department of Obstetrics and Gynaecology University of Melbourne and The Royal Women’s Hospital Victoria Australia

**Keywords:** Core outcomes, menopause, randomised clinical trials, vasomotor symptoms

## Abstract

**Background:**

There is substantial variation in how menopausal vasomotor symptoms are reported and measured among intervention studies. This has prevented meaningful comparisons between treatments and limited data synthesis.

**Objectives:**

To review systematically the outcome reporting and measures used to assess menopausal vasomotor symptoms from randomised controlled trials of treatments.

**Search strategy:**

We searched MEDLINE, Embase, and Cochrane Central Register of Controlled Trials from inception to May 2018.

**Selection criteria:**

Randomised controlled trials with a primary outcome of menopausal vasomotor symptoms in women and a sample size of at least 20 women per study arm.

**Data collection and analysis:**

Data about study characteristics, primary vasomotor‐related outcomes and methods of measuring them.

**Main results:**

The search identified 5591 studies, 214 of which were included. Forty‐nine different primary reported outcomes were identified for vasomotor symptoms and 16 different tools had been used to measure these outcomes. The most commonly reported outcomes were frequency (97/214), severity (116/214), and intensity (28/114) of vasomotor symptoms or a composite of these outcomes (68/214). There was little consistency in how the frequency and severity/intensity of vasomotor symptoms were defined.

**Conclusions:**

There is substantial variation in how menopausal vasomotor symptoms have been reported and measured in treatment trials. Future studies should include standardised outcome measures which reflect the priorities of patients, clinicians, and researchers. This is most effectively achieved through the development of a Core Outcome Set. This systematic review is the first step towards development of a Core Outcome Set for menopausal vasomotor symptoms.

**Tweetable summary:**

Menopausal hot flushes and night sweats have been reported in 49 different ways in clinical research. A core outcome set is urgently required.

## Tweetable summary

Menopausal hot flushes and night sweats have been reported in 49 different ways in clinical research. A core outcome set is urgently required.

## Introduction

There is general agreement that vasomotor symptoms (hot flushes and night sweats) are the most common and problematic menopausal symptom.^1^, ^2^ Vasomotor symptoms are also the leading patient priority for treatment.^3^ Oestrogen‐containing menopausal hormone therapy (MHT) is an effective treatment for menopausal vasomotor symptoms; however, use of MHT has fallen substantially following concerns about safety.^4^ There is a growing focus on the development and evaluation of nonpharmacological and nonhormonal treatments for vasomotor symptoms.^5^ In addition, MHT is contraindicated in women with a personal history of breast cancer who may report more severe vasomotor symptoms than women experiencing natural menopause.^6^ Enhanced understanding of the central mechanisms regulating vasomotor symptoms is driving the development and evaluation of novel targeted therapies,^7^ but the interpretation and implementation of these studies is hampered by lack of consensus about how vasomotor symptoms should be reported and measured. This limits the potential to compare treatments and to synthesise the evidence, which in turn compromises decision‐making by clinicians and patients.

Current National Institute for Health and Care Excellence (NICE) guidelines on the management of menopause^8^ highlight the need for greater standardisation of outcome reporting and measures for treatment trials in menopause, and the consequent difficulty in evidence synthesis. There is an urgent need to determine what outcomes are most important to patients, clinicians, and researchers in order to increase the relevance of future intervention studies and facilitate comparisons between treatments.^9^, ^10^ The Core Outcomes in Effectiveness (COMET) initiative is leading protocols for the development of Core Outcome Sets. These are well defined, condition‐specific, and feasible outcomes which should be included as a minimum set of outcomes in intervention studies.^11^ To advance the development of Core Outcome sets in women’s health,^12^ 80 editors of women’s health journals have formed a consortium to support the development, dissemination, and implementation of core outcome sets within the reproductive field (Core Outcomes in Women's and Newborn Health‐CROWN, http://www.crown-initiative.org).^13^

The Core Outcomes in Menopause (COMMA) initiative is an international consortium of clinicians, researchers, and consumers developing a Core Outcome Set for menopausal symptoms. Following a standardised process, we have first systematically reviewed all randomised controlled trials (RCTs) of interventions for menopausal vasomotor symptoms to determine what outcomes have been reported and how they have been measured. We will then repeat this process for vaginal symptoms at menopause. This information will then be used to inform a Delphi survey by clinicians, researchers, and patients to identify priorities for inclusion in the final Core Outcome Set.^12^

## Methods

### Study eligibility

We included all RCTs with a primary outcome of female menopausal vasomotor symptoms and a sample size of at least 20 women per study arm to minimise the likelihood of including feasibility or pilot studies^14^. We excluded studies that assessed menopausal vasomotor symptoms as a secondary outcome, quasi‐randomised studies, secondary analyses of previously published RCTs, conference abstracts of RCTs, observational, analytical, or diagnostic studies and feasibility/pilot studies. We also excluded studies primarily aiming to assess pharmacokinetics, the mechanism of drug action, or tolerability and intervention studies with no explicit sample size calculations.

### Search strategy

We searched MEDLINE, Embase, and Cochrane Central Register of Controlled Trials (CENTRAL) until May 2018. We hand‐searched the reference lists of the included trials or other keynote publications. Search terms included menopause, menopausal, menopausal symptoms, climacteric, hot flush or flash, night sweat, vasomotor, and a search filter for RCTs (Appendix S1). There was no language restriction.

### Data extraction

Two reviewers (W.W.L. and S.I.) independently assessed the studies using the predefined criteria described above. Disagreement was resolved by discussion with the steering committee. Full articles were obtained and data were extracted using a prespecified extraction sheet.

### Quality assessment

Jadad scoring was used for assessing the methodological quality of the included trials.^15^ The 5‐point validated scoring system assesses the following: whether the trial (1) was described as randomised (1 point), (2) used an appropriate method of randomisation (1 point), (3) was blinded (1 point), (4) used an appropriate method of blinding (1 point), and (5) accounted for all patients randomised (1 point); ≤2 points was considered low quality and ≥3 was considered medium to high quality.

The quality of describing and reporting the outcome was assessed using the 6‐point Management of Otitis Media with Effusion in Cleft Palate (MOMENT) scoring criteria,^16^ which has been used previously in the context of quality assessment of studies for the development of a core outcome set and a cut‐off of ≥4 to indicate a high‐quality trial. The following points were considered: whether the primary outcome was (1) clearly stated (1 point), (2) clearly defined (1 point); whether the secondary outcomes were (3) clearly stated (1 point), (4) clearly defined (1 point); (5) whether the authors explained the use of the outcomes they selected (1 point); (6) whether specific methods were used to enhance the quality of outcome measurement (1 point).

### Patient involvement

Patients were not involved in the stage of conducting the systematic review but they will be involved in the subsequent steps (Delphi and consensus meeting) of developing the core outcome set.

### Core outcomes

Core outcomes do not exist in this research field and were therefore not used in the systematic search. Our aim is to develop and disseminate core outcomes of menopausal symptoms and this systematic review is the first step of the process.

### Funding

This study was funded by an MRC postdoctoral fellowship (S.I., MR/N015177/1), NHMRC Practitioner Fellowship (MH, APP 1058935) and an NHMRC Principal Research Fellowship (G.D.M., APP1121844). The funders were not involved in any stage of conducting this systematic review.

## Results

**Figure 1:**
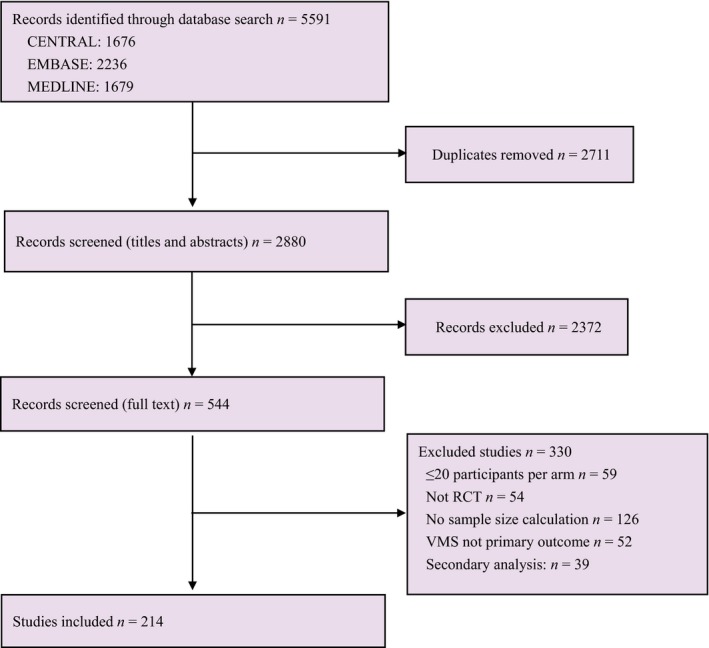
Flowchart of search strategy.

The search identified 5591 studies, of which 2711 duplicates were removed. We screened 2880 titles and abstracts and excluded 2372 records which did not meet the inclusion criteria. In all, 544 studies were read in full. Of these, 330 were excluded; 59 included fewer than 20 women per study arm and 54 were not an RCT; 126 did not clearly state a sample size calculation; 52 did not measure vasomotor symptoms as the primary outcome; 39 were secondary analysis. Following these exclusions, 214 RCT were included^17^, ^18^, ^19^, ^20^, ^21^, ^22^, ^23^, ^24^, ^25^, ^26^, ^27^, ^28^, ^29^, ^30^, ^31^, ^32^, ^33^, ^34^, ^35^, ^36^, ^37^, ^38^, ^39^, ^40^, ^41^, ^42^, ^43^, ^44^, ^45^, ^46^, ^47^, ^48^, ^49^, ^50^, ^51^, ^52^, ^53^, ^54^, ^55^, ^56^, ^57^, ^58^, ^59^, ^60^, ^61^, ^62^, ^63^, ^64^, ^65^, ^66^, ^67^, ^68^, ^69^, ^70^, ^71^, ^72^, ^73^, ^74^, ^75^, ^76^, ^77^, ^78^, ^79^, ^80^, ^81^, ^82^, ^83^, ^84^, ^85^, ^86^, ^87^, ^88^, ^89^, ^90^, ^91^, ^92^, ^93^, ^94^, ^95^, ^96^, ^97^, ^98^, ^99^, ^100^, ^101^, ^102^, ^103^, ^104^, ^105^, ^106^, ^107^, ^108^, ^109^, ^110^, ^111^, ^112^, ^113^, ^114^, ^115^, ^116^, ^117^, ^118^, ^119^, ^120^, ^121^, ^122^, ^123^, ^124^, ^125^, ^126^, ^127^, ^128^, ^129^, ^130^, ^131^, ^132^, ^133^, ^134^, ^135^, ^136^, ^137^, ^138^, ^139^, ^140^, ^141^, ^142^, ^143^, ^144^, ^145^, ^146^, ^147^, ^148^, ^149^, ^150^, ^151^, ^152^, ^153^, ^154^, ^155^, ^156^, ^157^, ^158^, ^159^, ^160^, ^161^, ^162^, ^163^, ^164^, ^165^, ^166^, ^167^, ^168^, ^169^, ^170^, ^171^, ^172^, ^173^, ^174^, ^175^, ^176^, ^177^, ^178^, ^179^, ^180^, ^181^, ^182^, ^183^, ^184^, ^185^, ^186^, ^187^, ^188^, ^189^, ^190^, ^191^, ^192^, ^193^, ^194^, ^195^, ^196^, ^197^, ^198^, ^199^, ^200^, ^201^, ^202^, ^203^, ^204^, ^205^, ^206^, ^207^, ^208^, ^209^, ^210^, ^211^, ^212^, ^213^, ^214^, ^215^, ^216^, ^217^, ^218^, ^219^, ^220^, ^221^, ^222^, ^223^, ^224^, ^225^, ^226^, ^227^, ^228^, ^229^, ^230^ (Figure 1). Table S1 describes the 214 studies with a total of 22 682 participants. The studies were published between 1994 and 2018. More than one‐third of the trials (77/214, 36%) were conducted in USA. The follow‐up period ranged from 4 to 52 weeks, with half the included studies following up participants for 12 weeks (108/214, 50%).

### Reported outcomes

The included trials reported 31 different interventions for menopausal vasomotor symptoms: 68 (32%) included hormone therapies and the remaining 146 nonhormonal therapies; 46 were prescription therapies; and 100 were nonprescription therapies. Among these interventions, estrogen and/or progestogen therapies (61/214, 29%) and over‐the‐counter dietary or herbal supplements (68/214, 32%) were the most common interventions (Table 1).

**Table 1:** Interventions for menopausal vasomotor symptoms

Interventions	Trials, *n* (%)
**Hormone therapy**	**68 (32)**
Estrogen alone	45
Estrogen + progestogen	12
Estrogen + bazedoxifene	1
Progestogen alone	4
Tibolone	6
**Nonhormonal therapy**	
Prescription therapies	**46 (21)**
SSRIs/SNRIs	26
Antiepileptic	10
ERr 731	2
BRN‐01	1
Cinnarizin	1
Gamolenic acid	1
L‐isoleucine	1
MF 101	1
Neurokinin 3	1
Oxybutynin	1
RAD 1901	1
Nonprescription therapies	**100 (47)**
Over‐the‐counter dietary and/or herbal products	68
Acupuncture	8
Exercise	5
Lifestyle education	4
Relaxation	3
Paced respiration	3
Cognitive therapy/cognitive behaviour therapy	4
Mindfulness training	2
Guasha	1
Local thermal therapy	1
Clinical hypnosis	1
Total RCTs	**214**

MF 101, Menopausal Formula 101; RCTs, randomised controlled trials; SSRIs, selective serotonin reuptake inhibitors; SNRIs, serotonin and norepinephrine reuptake inhibitors.

ERr 731: A special extract from the roots of *Rheum rhaponticum*, referred to as ERr 731 (tradename Phytoestrol N)

BRN‐01: A homeopathic medicine registered in France for menopausal hot flushes, combining the five homeopathic medications: *Actaea racemosa* (4 centesimal dilutions [4CH]), *Arnica montana* (4CH), *Glonoinum* (4CH), *Lachesis mutus* (5CH), and *Sanguinaria canadensis* (4CH).

RAD1901: An orally available, selective estrogen receptor degrader (SERD) and selective estrogen receptor modulator (SERM).

Forty‐nine primary outcomes were identified from 214 RCTs including 22 682 women. Almost half of the RCTs (94/214, 44%) only included postmenopausal women, 12% (26/214) only included women with a history of breast cancer, and 5% (12/214) included peri‐ and postmenopausal women. Around a quarter of the RCTs (56/214, 26%) included both surgical and naturally peri‐ and postmenopausal women. We categorised the primary outcomes into four domains: (1) purely vasomotor‐related outcomes (183/214, 86%); (2) quality‐of‐life‐related outcomes (9/214, 4%); (3) composite outcomes (17/214, 8%); and (4) functional impact, specifically how bothersome, interfering and problematic vasomotor symptoms are; for this review we will refer to the latter category as 'interference' outcomes (5/214, 2.3%). The largest group was purely vasomotor‐related outcomes, comprising 33 individual outcomes. The second largest group was composite outcomes, all of which included vasomotor symptoms as one of the parameters. Nine trials assessed quality of life^28^, ^77^, ^98^, ^122^, ^135^, ^163^, ^181^, ^197^, ^228^ and five trials assessed interference^33^, ^60^, ^66^, ^92^, ^138^ as primary outcomes (Table 2).

**Table 2:** Vasomotor‐related outcome categories

Outcome categories	The number of ways the outcomes is expressed	Trials, *n* (%)
**Purely vasomotor symptoms** Frequency of HF Frequency of HF/NS Frequency of moderate to severe HF Frequency of moderate to severe HF/NS Number of HF Number of HF/NS Number of moderate to severe HF Number of severe HF/NS Severity of HF Severity of HF/NS Severity of moderate to severe HF Severity of moderate to severe HF/NS Intensity of HF Intensity of HF/NS Incidence of HF HF (composite/severity) score 41% reduction in HF 44% reduction in HF 50% reduction in HF 75% reduction in HF Frequency of awakenings resulting from nocturnal vasomotor symptoms More than 50% patients halved the distress of HF/NS Moderate to severe rate of HF Percentage of HF reported Proportion of patients responding about vasomotor symptoms Vasomotor complaints Percentage change in HF score Vasomotor symptoms (assessed with the Blatt‐Kupperman Index) HF (assessed with the Greene climacteric) Sweating at night assessed with the Greene climacteric) Vasomotor symptoms per day (HF and NS, assessed with the Wiklund scale) Vasomotor symptom intensity (assessed with the Wiklund scale) Simplified Menopausal Index score	**33**	**177 (83)**
**Quality of life (QOL)**	**4**	**9 (4)**
**Interference**	**5**	**11 (5)**
The extent HF/NS regarded as problem during last week How distressed one feels about HF during last week How much HF interfered with daily routine over the last week Bothersomeness of HF/NS Perceived perimenopausal disturbances scale score		
**Composite**	**7**	**17 (8)**
VMS + QOL		5
VMS + urogenital symptoms		4
VMS + sleep quality		2
VMS + side‐effect		3
VMS + endocrine symptoms		1
VMS + pharmacodynamic markers		1
VMS + QOL + satisfaction		1
**Total RCTs**		**214**

HF, Hot flushes; NS, night sweats; QoL, quality of life; VMS, vasomotor symptoms.

### Measurement tools

Seven different measurement tool categories were used to measure purely vasomotor‐related outcomes. Most (158/214, 74%,) included trials used diary records of vasomotor symptoms and 24.7% (53/214) used menopause‐specific subscales. Of these subscales, the Kupperman Menopausal Index (25/57, 44%), Greene Climacteric Scale (15/57, 26%), and Menopause Rating Scale (MRS) (10/57, 18%) were the three most frequently used measurement tools. The Hot Flush Rating Scale (HFRS)^33^, ^60^, ^66^, ^92^, ^138^ measures how vasomotor symptoms interfere with daily routine and activities^231^, ^232^. The Hot Flush Related Daily Interference scales (HFRDIS) and the shortened Hot Flush Interference (HFI) scale^233^, ^234^ have been used to measure interference due to vasomotor symptoms but were not the primary outcome of eligible RCTs, and so were not included in the systematic review. However, they will still be used for the subsequent Delphi process. Other subjective vasomotor symptoms measurement tools included a 20‐item structured symptom checklist; two of the items asking about the presence of hot flushes and cold/night sweats,^128^ a 5‐point (from none to very severe) scoring system about the severity of hot flushes and night sweats,^103^ Interactive Voice Response System to record the number and severity of the hot flushes,^157^ and self‐reported surveys.^156^ Objective measures of vasomotor symptoms such as skin conductance were used in five trials in addition to subjective measures^33^, ^68^, ^118^, ^138^, ^167^ (Table 3).

**Table 3:** Tools for measuring vasomotor‐related outcomes

Tools	*n*
**Purely vasomotor symptoms**	
Hot flushes diary/electronic diary	158
Menopausal‐specific scale	53
Kupperman Menopausal Index (KMI)	25
Greene Climacteric Scale (GCS)	15
Menopause Rating Scale (MRS)	10
Wiklund Vasomotor Symptom Subscale score	1
Perceived Perimenopausal Disturbances Scale	1
Simplified Menopausal Index (SMI)	1
Structured menopausal‐specific checklist	1
Skin conductance monitor system	5
Interactive voice system	1
Symptoms scoring system	1
Self‐reported validated survey instruments	1
**Quality of life**	15
MENQOL	12
WHQ	2
EORTC QLQ‐C30	1
**Interference**	
The Hot Flush Rating Scale (HFRS)	5

Diaries were used to record 25 different types of menopausal vasomotor‐related outcomes and accounted for 57% (25/44) of all primary outcomes. The number, frequency, severity, and intensity of menopausal vasomotor symptoms were the most commonly reported vasomotor‐related outcomes assessed by diaries. However, there was substantial variation in the definitions of each outcome. For example, for frequency, the majority of included studies (72/214) reported the ‘number of vasomotor symptoms per 24 h’ (8 retrospectively and 64 prospectively), and 39/214 measured the ‘number of vasomotor symptoms per week’. Table S2 shows the complete list of vasomotor‐related outcomes recorded by diaries and how often they have been reported in RCTs of intervention studies for vasomotor symptoms.

Quality of life outcomes was measured by three scales: Menopausal‐Specific Quality of Life (MENQOL),^28^, ^77^, ^90^, ^98^, ^109^, ^122^, ^130^, ^141^, ^181^, ^197^, ^198^, ^228^ Women’s Health Questionnaire (WHQ),^135^, ^163^ and the European Organisation for Research and Treatment of Cancer Quality of Life‐Care30 (EORTC QLQ‐C30).^136^ Of these, 14 of 15 trials chose menopausal‐specific scales (MENQOL, WHQ). Only one trial including women with vasomotor symptoms after breast cancer used a general QOL scale (EORTC QLQ‐C30)^136^ (Table 3).

### Variation in the definitions of vasomotor‐related outcomes

There was substantial heterogeneity in the definition of vasomotor‐related outcomes. Three different definitions were used to measure the frequency of vasomotor symptoms. Most studies (79/97, 81%) defined frequency as the number of hot flushes or night sweats, whereas 18 studies did not define how frequency was measured. The severity of vasomotor symptoms was defined in nine different ways and the intensity in seven different ways (Table S3). The 68 studies reporting composite outcomes for vasomotor symptoms utilised 11 different ways of defining the composite score. The most commonly used approach (27/68, 40%) measured the number of hot flushes and night sweats, and calculated a composite score weighted by severity rating. There was considerable overlap between composite score definitions.

### Quality assessment of trials

Regarding methodological quality, 34% (73/214) of included RCTs scored 5 out of 5 points on the Jadad scale. More than half of the trials (118/214, 55%) scored 6 out of 6 points on the MOMENT scale (Table S1).

## Discussion

### Main findings

This is the first systematic review of outcomes used to measure vasomotor symptoms in randomised controlled trials of interventions. Our findings demonstrate major inconsistencies in how treatments for the same symptoms have been evaluated. For example, the severity of hot flushes and night sweats had nine different definitions. Overall, the most commonly used outcomes for vasomotor symptoms (based on 214 RCTs including 22 682 women) were the frequency and intensity of vasomotor symptoms or a compound measure of these (*n* = 59). A smaller number of studies (*n* = 5) took a different approach and measured the interference due to vasomotor symptoms. It remains unclear which measures of vasomotor symptoms best reflect the priorities of patients, clinicians, and researchers, and this will be addressed by the development of a Core Outcome Set. Inclusion of the Core Outcome Set in future intervention studies for vasomotor symptoms will enhance the quality and relevance of trials and facilitate decision‐making by clinicians and patients.

### Strengths and limitations

We conducted a comprehensive search strategy with a robust methodological design to include all large (>20 participants per arm) RCTs of interventions for vasomotor symptoms. Two researchers independently evaluated the available evidence to minimise overlooking relevant evidence. To our knowledge, this is the first time that reported vasomotor‐related outcomes have been synthesised, a necessary step to inform the Delphi process for key stakeholders to rate the components of the core outcome set.^12^ Although most included trials were of medium or high methodological standard, the diverse nature of the outcome measures used diminishes the value of these trials to inform patient choices and clinical decision‐making.

This study focused on menopausal vasomotor symptoms in the first instance. We recognise that personal, ethnic, cultural, and geographical factors influence the nature and experience of menopause, and that not all women experience vasomotor symptoms at menopause.^235^ We comprehensively searched three major databases and it is unlikely we have missed an RCT published elsewhere. We acknowledge we did not search CINAHL, but we doubt that additional RCTs could only be identified in that database. We included randomised trials with vasomotor‐related symptoms as a primary outcome with over 20 participants in each arm, excluding observational studies and pilot studies. However, given the large number of included studies, we do not anticipate that we have missed outcomes not captured in larger trials. We recognise that this systematic review was limited to trials where vasomotor symptoms were the primary outcome. However, given the large number of studies included, we do not considered that we missed important outcomes. The expert panel and Delphi process will highlight any additional important outcomes that may have been overlooked because they were not included in treatment studies or were reported as secondary outcomes. We also appreciate that our findings may be skewed towards FDA‐driven outcomes, as many studies were conducted in USA. We have only listed the range of outcome measures used and have not applied any qualitative assessment of the value or importance of these measures for women or clinicians. Only a few relatively recent RCTs have measured the impact of interventions on the interference caused in women by vasomotor symptoms and it is uncertain whether the frequency/severity or interference of symptoms best reflects women’s treatment priorities. These issues will be addressed by the Delphi survey and the subsequent consensus meeting. Most published RCTs of interventions for vasomotor symptoms focused on caucasian women who may experience menopause differently from other ethnicities.^236^ The COMMA consortium includes representation from a wide range of geographical areas and ethnic groups to ensure that the Core Outcome Set reflects variations in stakeholder priorities.

## Interpretation

Inconsistency in measures used for the evaluation of treatments for vasomotor symptoms limits comparisons between treatments and the interpretation of findings for clinical practice.^237^ Understanding the efficacy of new treatments and how they compare with existing approaches requires the use of standardised outcome measures that are meaningful to patients, and feasible for clinicians and researchers.^238^

A Core Outcome Set does not preclude the inclusion of additional outcome measures, but sets a minimum standard of outcomes that should be reported in all interventional trials. This systematic review is the first step towards the development of meaningful consensus by identifying how vasomotor symptoms have been measured to inform consensus through the Delphi process.^239^

## Conclusion

Most intervention studies for vasomotor symptoms have measured frequency or severity of symptoms, or a combination of both. Some have measured the interference caused in daily life due to symptoms. There is a need for consensus around the optimum outcomes and how these should be measured to facilitate comparisons between interventions and ensure patient‐centred clinical practice.

### Disclosure of interests

No conflict of interest to disclose. Completed disclosure of interest forms are available to view online as supporting information.

### Contribution of authorship

MH and SI conceived the idea and set the protocol. GM, RN, and MAL refined the protocol. SI and WW conducted the systematic search, and SI wrote the first draft of the paper with contribution from WW in writing up the methods. MSH provided useful insight regarding the bothersome aspect of menopausal symptoms. All authors edited and accepted the manuscript prior to submission.

### Details of ethics approval

No ethics approval was required as we have summarised already published data.

### Funding

This study was funded by an MRC postdoctoral fellowship (S.I., MR/N015177/1), NHMRC Practitioner Fellowship (M.H., APP 1058935), and an NHMRC Principal Research Fellowship (G.D.M., APP1121844). The funders were not involved in any stage of this systematic review.

## Supporting information


**Table S1.** Study characteristics and quality assessment scoring of the included studies.

**Table S2.** Vasomotor‐related outcomes measured by diary.

**Table S3.** Different definitions for frequency, severity, intensity, and vasomotor scores of vasomotor‐related outcomes.

**Appendix S1.** Search strategy.
