# Supplementary material for: Variation in menopausal vasomotor symptoms outcomes in clinical trials: a systematic review
Source: BJOG. 2019 Nov 13;127(3):320–33. doi: 10.1111/1471-0528.15990 (PMC6972542; doi:10.1111/1471-0528.15990)
Supplement: Supplementary file 1 — Table S1. Study characteristics and quality assessment scoring of the included studies. [file BJO-127-320-s001.pdf]

Table S1. Study characteristics and quality assessment scoring of the included studies

Author	Year	Title	Journal	Country	Population	Participants	Follow-up duration	Intervention	Jadad Score (max 5)	MOMENT Score (max 6)
Aghamiri, V.	2016	The effect of Hop (Humulus lupulus L.) on early menopausal symptoms and HF: A randomized placebo-controlled trial	Complementary Therapies in Clinical Practice	Iran	pre and postmenopausal	120	12 wks	dietary supplements	5	2
Aguirre, W	2010	Gabapentin vs. low-dose transdermal estradiol for treating post-menopausal women with moderate to very severe HF	Gynecological Endocrinology	Ecuador	postmenopausal	45	8 wks	Antiepileptics	2	2
Al-Akoum, M.	2009	Effects of Hypericum perforatum (St. John's wort) on HF and quality of life in perimenopausal women: a randomized pilot trial	Menopause	Canada	perimenopausal	47	12 wks	dietary supplements	4	4
Al-Azzawi, F. and H. Buckler	2003	Comparison of a novel vaginal ring delivering estradiol acetate versus oral estradiol for relief of vasomotor menopausal symptoms	Climacteric	UK	postmenopausal and surgical	159	24 wks	HRT vaginal ring	5	4
Allameh, Z.	2013	Comparison of Gabapentin with Estrogen for treatment of HF in post-menopausal women.	Journal of Research in Pharmacy Practice	Iran	postmenopausal	100	12 wks	Antiepileptics	1	3
Anderson, D. J.	2015	Facilitating lifestyle changes to manage menopausal symptoms in women with breast cancer: a randomized	Menopause	Australia	breast cancer	55	12 wks	education	3	6

		controlled pilot trial of The Pink Women's Wellness Program.								
Andrikoula, M.	2011	The effects of micronutrient supplementation on vasomotor symptoms in postmenopausal women. C	Climacteric	UK	postmenopausal	91	14 wks	dietary supplements	5	2
Archer, D. F., et al.	2003	Percutaneous 17beta-estradiol gel for the treatment of vasomotor symptoms in postmenopausal women.	Menopause	USA	postmenopausal	221	12 wks	transdermal HRT	4	2
Archer, D. F., et al.	2009	Desvenlafaxine for the treatment of vasomotor symptoms associated with menopause: a double-blind, randomized, placebo-controlled trial of efficacy and safety.	American Journal of Obstetrics and Gynecology	USA	postmenopausal	567	26 wks	SSRI	4	2
Archer, D., et al.	2009	A double-blind, randomly assigned, placebo-controlled study of desvenlafaxine efficacy and safety for the treatment of vasomotor symptoms associated with menopause.	American Journal of Obstetrics and Gynecology	USA	postmenopausal and surgical	458	12 wks	SSRI	5	6
Archer, D., et al.	2014	A randomized, double-blind, placebo-controlled study of the lowest effective dose of drospirenone with 17?-estradiol for moderate to severe vasomotor symptoms in	Menopause	USA	postmenopausal and surgical	735	12 wks	oral HRT	5	6

		postmenopausal women.								
Asghari, M.	2017	Effect of aerobic exercise and nutrition educationon quality of life and early menopause symptoms:A randomized controlled trial	Women & Health	Iran	pre and postmenopausal	54	12 wks	exercise	3	2
Aslan, E., et al.	2007	How best is to discontinue postmenopausal hormone therapy: immediate or tapered?	Maturitas	Turkey	postmenopausal	70	4 wks	oral HRT	2	4
Aso, T.	2012	A natural S-equol supplement alleviates HF and other menopausal symptoms in equol nonproducing postmenopausal Japanese women	Journal of Women's Health	Japan	postmenopausal	160	12 wks	S-equol	5	2
Auerbach, L.	2012	Pomegranate seed oil in women with menopausal symptoms: a prospective randomized, placebo-controlled, double-blinded trial	Menopause	Austria	postmenopausal	80	12 wks	dietary supplements	3	2
Avis, N., et al.	2016	Acupuncture in Menopause (AIM) study: a pragmatic, randomized controlled trial.	Menopause	USA	peri and postmenopausal	209	12 mos	acupuncture	2	6
Ayers, B.	2012	Effectiveness of group and self-help cognitive behavior therapy in reducing problematic menopausal HF and NS (MENOS 2): A	Menopause	USA	peri and postmenopausal	140	26 wks	cognitive therapy	3	6

		randomized controlled trial								
Bacchi-Modena, A	1997	Efficacy and tolerability of (R)Estraderm MX, a new estradiol matrix patch	Maturitas	Italy	postmenopausal and surgical	177	12 wks	transdermal HRT	3	6
Bachmann, G., et al.	2007	Lowest effective transdermal 17beta-estradiol dose for relief of HF in postmenopausal women: a randomized controlled trial.	Obstetrics and Gynecology	USA	postmenopausal and surgical	425	12 wks	transdermal HRT	5	6
Bachmann, G., et al.	2011	Effects of bazedoxifene in nonflushing postmenopausal women: a randomized phase 2 trial.	Menopause	USA	postmenopausal	494	12 wks	SSRI	5	6
Bai, W.	2007	Efficacy and tolerability of a medicinal product containing an isopropanolic black cohosh extract in Chinese women with menopausal symptoms: a randomized, double blind, parallel-controlled study versus tibolone	Maturitas	China	postmenopausal	244	12 wks	dietary supplements	4	6
Barton, D. L.	2010	Phase III, placebo-controlled trial of three doses of citalopram for the treatment of HF: NCCTG trial N05C9	Journal of Clinical Oncology	USA	breast cancer	254	6 wks	SSRI	1	6
Benster, B., et al	2009	A double-blind placebo-controlled study to evaluate the effect of progestelle progesterone cream on postmenopausal women	Menopause International	UK	postmenopausal and surgical	230	6 mos	transdermal HRT	4	6

Bertelli, G	2002	Intramuscular depot medroxyprogesterone versus oral megestrol for the control of postmenopausal HF in breast cancer patients: a randomized study	Annals of Oncology	Italy	breast cancer	71	6 wks	oral HRT	2	4
Biglia, N., et al.	2009	Non-hormonal treatment of HF in breast cancer survivors: gabapentin vs. vitamin E	Climacteric	Italy	breast cancer	115	12 wks	Antiepileptics	3	1
Bokmand, S.	2003	Acupuncture relieves menopausal discomfort in breast cancer patients: a prospective, double blinded, randomized study	Breast	Finland	breast cancer	94	12 wks	acupuncture	3	2
Borud, E., et al.	2009	The Acupuncture on HF Among Menopausal Women (ACUFLASH) study, a randomized controlled study	Menopause	Norway	postmenopausal	276	12 wks	acupuncture	2	6
Bouchard, P., et al.	2012	Randomized placebo- and active-controlled study of desvenlafaxine for menopausal vasomotor symptoms.	Climacteric	35 sites in Europe, two sites in South Africa, and one site in Mexico.	postmenopausal	485	12 wks	SSRI	3	6
Burke, G., et al	2003	Soy protein and isoflavone effects on vasomotor symptoms in peri- and postmenopausal women: the Soy Estrogen Alternative Study.	Menopause	USA	peri and postmenopausal	241	12 wks	isoflavone	3	4
Buster, J., et al.	2008	Low-dose estradiol spray to treat vasomotor symptoms:	Obstetrics and Gynecology	USA, 43 sites	postmenopausal and surgical	454	12 wks	transdermal HRT	4	4

		a randomized controlled trial.								
Butt, D., et al.	2008	Gabapentin for the treatment of menopausal HF: a randomized controlled trial.	Menopause	Canada, greater Toronto area	postmenopausal	197	4 wks	Antiepileptics	4	2
Carmignani, L. O.	2010	The effect of dietary soy supplementation compared to estrogen and placebo on menopausal symptoms: a randomized controlled trial	Maturitas	Brazil	postmenopausal	60	16 wks	isoflavone	5	2
Carmody, J. F.	2011	Mindfulness training for coping with HF: results of a randomized trial	Menopause	USA	peri and postmenopausal	110	20 wks	mindfulness training	3	6
Carpenter, J. S	2012	Effect of escitalopram on HF interference: a randomized, controlled trial.	Fertility & Sterility	USA	postmenopausal and surgical	205	4 wks	SSRI	5	6
Carpenter, J., et al.	2013	Paced respiration for vasomotor and other menopausal symptoms: a randomized, controlled trial.	Journal of General Internal Medicine	USA, Midwestern city and surrounding area	breast cancer and postmenopausal	218	16 wks	respiration	2	6
Cezarino, P. Y	2011	The effects of cinnarizine on menopausal symptoms in women	Climacteric	Brazil	postmenopausal	100	6 mos	cinnarizin	5	1
Chandeying, V. and M. Sangthawan	2007	Efficacy comparison of Pueraria mirifica (PM) against conjugated equine estrogen (CEE) with/without medroxyprogesterone acetate (MPA) in the treatment of climacteric symptoms	Journal of the Medical Association of Thailand	Thailand	perimenopausal	71	6 mos	dietary supplements	1	6

		in perimenopausal women: phase III study								
Chang, A., et al.	2012	The effect of herbal extract (EstroG-100) on pre-, peri- and post-menopausal women: a randomized double-blind, placebo-controlled study	Phytotherapy research : PTR	Korea	pre-peri-postmenopausal	64	12 wks	oral HRT	5	3
Chenoy, R.	1994	Effect of oral gamolenic acid from evening primrose oil on menopausal flushing	BMJ	UK	postmenopausal	56	6 mos	gamolenic acid	2	2
Chien, L., et al.	2011	Local thermal therapy effects on menopausal symptoms and bone mineral density.	Journal of alternative and complementary medicine (new york, N.Y.)	China, Taiwan	postmenopausal	47	1o wks	local thermal therapy	1	2
Chung, D. J.	2007	Black cohosh and St. John's wort (GYNO-Plus) for climacteric symptoms.	Yonsei Medical Journal	Korean	perimenopausal	89	12 wks	dietary supplements	4	5
Cohen, L., et al.	2014	Efficacy of omega-3 for vasomotor symptoms treatment: a randomized controlled trial.	Menopause	USA	peri and postmenopausal and surgical	355	12 wks	dietary supplements	3	6
Colau, J. C	2012	Efficacy of a non-hormonal treatment, BRN-01, on menopausal HF: a multicenter, randomized, double-blind, placebo-controlled trial	Drugs in R & D	France	postmenopausal	108	12 wks	BRN-01	5	6
Cortés-Bonilla, M	2015	Treatment of menopausal symptoms with three low-dose continuous sequential 17?-estradiol/progesterone parenteral monthly	Gynecological endocrinology	Mexico	peri and postmenopausal	103	6 mos	oral HRT	3	6

		formulations using novel non-polymeric microsphere technology								
Daley, A., et al.	2015	The effectiveness of exercise as treatment for vasomotor menopausal symptoms: randomised controlled trial	BJOG	UK, West Midlands	peri and postmenopausal	261	6 mos	exercise	2	6
Davinelli, S.	2017	Influence of equol and resveratrol supplementation on health-related quality of life in menopausal women: A randomized, placebo-controlled study	Maturitas	Italy	postmenopausal	60	12 wks	S-equol	5	2
Davis, S. R.,	2017	The effects of Chinese medicinal herbs on postmenopausal vasomotor symptoms of Australian women. A randomised controlled trial.	Medical Journal of Australia	Australia	postmenopausal	78	12 wks	dietary supplements	5	6
de Luca, A. C	2011	Acupuncture-ameliorated menopausal symptoms: single-blind, placebo-controlled, randomized trial	Climacteric	Brazil	postmenopausal	80	12 wks	acupuncture	3	2
de Vrijer, B.	2000	Efficacy and tolerability of a new estradiol delivering matrix patch (Estraderm MX) in postmenopausal women	Maturitas	Netherlands	postmenopausal and surgical	254	12 wks	transdermal HRT	4	6
del Giorno, C	2010	Effects of Trifolium pratense on the climacteric and sexual symptoms in	Revista Da Associacao Medica Brasileira	Brazil	postmenopausal	120	12 mos	dietary supplements	5	2

		postmenopause women								
Duijts, S. F	2012	Efficacy of cognitive behavioral therapy and physical exercise in alleviating treatment-induced menopausal symptoms in patients with breast cancer: results of a randomized, controlled, multicenter tria	Journal of Clinical Oncology	Netherlands	breast cancer	422	12 wks	cognitive behavioral treatment	3	5
Ee, C.	2016	Acupuncture for Menopausal HF: A Randomized Trial	Annals of Internal Medicine	Australia	peri and postmenopausal and surgical	127	8 wks	acupuncture	5	4
Elkins, G. R	2012	Clinical hypnosis in the treatment of postmenopausal HF: A randomized controlled trial	Menopause	USA, Texas	postmenopausal and surgical	187	12 wks	clinical hypnosis	2	6
Endrikat, J	2007	A multicenter, prospective, randomized, double-blind, placebo-controlled study to investigate the efficacy of a continuous-combined hormone therapy preparation containing 1mg estradiol valerate/2mg dienogest on HF in postmenopausal women	Maturitas	Germany	postmenopausal and surgical	324	12 wks	oral HRT	2	4
Evans, M.	2005	Management of postmenopausal HF with venlafaxine hydrochloride: a randomized, controlled trial	Obstetrics & Gynecology	USA	postmenopausal and surgical	80	12 wks	SSRI	5	2

Evans, M.,	2011	The effect of synthetic genistein on menopause symptom management in healthy postmenopausal women: a multi-center, randomized, placebo-controlled study	Maturitas	Canada	postmenopausal and surgical	84	12 wks	isoflavone	5	2
Faure, E. D.	2002	Effects of a standardized soy extract on HF: a multicenter, double-blind, randomized, placebo-controlled study	Menopause	France	postmenopausal	75	4 mos	isoflavone	4	4
Fenlon, D. R.,	2008	A randomized controlled trial of relaxation training to reduce HF in women with primary breast cancer	Journal of Pain & Symptom Management	UK	breast cancer	150	3 mos	relaxation	3	2
Ferrari, A.	2009	Soy extract phytoestrogens with high dose of isoflavones for menopausal symptoms	Journal of Obstetrics & Gynaecology Research	Italy	postmenopausal	180	12 wks	isoflavone	5	6
Freeman, E. W., et al.	2011	Efficacy of escitalopram for HF in healthy menopausal women: a randomized controlled tria	JAMA	USA	peri and postmenopausal and surgical	205	8 wks	SSRI	5	6
Frei-Kleiner, S.,	2005	Cimicifuga racemosa dried ethanolic extract in menopausal disorders: a double-blind placebo-controlled clinical trial	Maturitas	Switzerland	peri and postmenopausal	122	12 wks	dietary supplements	4	6
Fu, S., et al	2016	A randomized, double-blind, placebo-controlled trial of Chinese herbal	Menopause	China, Tianjin	peri and postmenopausal	398	12 wks	dietary supplements	4	4

		medicine granules for the treatment of menopausal symptoms by stages								
Garcia, J.	2010	Use of a multibotanical (Nutrafem) for the relief of menopausal vasomotor symptoms: a double-blind, placebo-controlled study	Menopause		Singapore	postmenopausal	159	12 wks	dietary supplements	4	6
Gelfand, M. M., et al.	2003	Clinical assessment and quality of life of postmenopausal women treated with a new intermittent progestogen combination hormone replacement therapy: a placebo-controlled study.	Menopause		Canada	postmenopausal	119	90 d	oral HRT	3	4
Geller, S., et al.	2009	Safety and efficacy of black cohosh and red clover for the management of vasomotor symptoms: a randomized controlled trial.	Menopause		USA	peri and postmenopausal	89	12 mos	dietary supplements	5	6
Good, W. R	1999	Comparison of Alora estradiol matrix transdermal delivery system with oral conjugated equine estrogen therapy in relieving menopausal symptoms. Alora Study Group	Climacteric		USA	postmenopausal and surgical	321	12 wks	transdermal HRT	2	6
Good, W. R.	1996	Double-masked, multicenter study of an estradiol matrix transdermal delivery	Clinical Therapeutics		USA	postmenopausal and surgical	273	12 wks	transdermal HRT	2	6

		system (Alora) versus placebo in postmenopausal women experiencing menopausal symptoms. Alora Study Group								
Goodwin, J., et al.	2008	Phase III randomized placebo-controlled trial of two doses of megestrol acetate as treatment for menopausal symptoms in women with breast cancer: southwest Oncology Group Study	with T1-3, N0-1, M0 breast cancer	USA	breast cancer	88	6 mos	oral HRT	3	6
Gordon, P. R	2006	Sertraline to treat HF: a randomized controlled, double-blind, crossover trial in a general population	Menopause	USA	postmenopausal	102	4 weeks	SSRI	4	2
Grady, D.	2007	Ineffectiveness of sertraline for treatment of menopausal HF: a randomized controlled trial	Obstetrics & Gynecology	USA	peri and postmenopausal	99	6 wks	SSRI	4	6
Grady, D.	2009	MF101, a selective estrogen receptor beta modulator for the treatment of menopausal HF: a phase II clinical trial	Menopause	USA	postmenopausal and surgical	217	12 wks	MF 101	4	6
Guttuso, T.	2003	Gabapentin's effects on HF in postmenopausal women: a randomized controlled trial	Obstetrics & Gynecology	USA	postmenopausal and surgical	59	12 wks	Antiepileptics	4	6
Guttuso, T.,	2008	Effects of L-isoleucine and L-valine on HF and serum homocysteine: a randomized controlled trial	Obstetrics & Gynecology	USA	postmenopausal	86	12 wks	L-isoleucine	5	6

Haimov-Kochman, R., et al.	2006	Gradual discontinuation of hormone therapy does not prevent the reappearance of climacteric symptoms: a randomized prospective study	Menopause	Hongkong	postmenopausal	91	12 mos	oral HRT	1	2
Haines, C., et al.	2009	Micro-dose transdermal estradiol for relief of HF in postmenopausal Asian women: a randomized controlled trial.	Climacteric	Thailand, the Philippines, Singapore, Hong Kong and Malaysia.	postmenopausal and surgical	65	12 wks	oral HRT	4	6
Haines, C., et al.	2008	A randomized, double-blind, placebo-controlled study of the effect of a Chinese herbal medicine preparation (Dang Gui Buxue Tang) on menopausal symptoms in Hong Kong Chinese women	Climacteric	Hongkong	postmenopausal	103	12 wks	dietary supplements	5	4
Hardy, C.	2018	Self-help cognitive behavior therapy for working women with problematic HF and NS (MENOS@Work): a multicenter randomized controlled trial	Menopause	UK	peri and postmenopausal and surgical	124	20 wks	cognitive therapy	3	6
Hattersley, G., et al.	2017	Clinical investigation of RAD1901, a novel estrogen receptor ligand, for the treatment of postmenopausal vasomotor symptoms:	Menopause	USA	postmenopausal and surgical	100	4 wks	RAD 1901	4	5

		a phase 2 randomized, placebo-controlled								
Hedrick, R. E., et al	2009	Transdermal estradiol gel 0.1% for the treatment of vasomotor symptoms in postmenopausal women	Menopause	USA	postmenopausal and surgical	488	12 wks	transdermal HRT	2	6
Heger, M.,	2006	Efficacy and safety of a special extract of Rheum rhaponticum (ERr 731) in perimenopausal women with climacteric complaints: a 12-week randomized, double-blind, placebo-controlled trial	Menoppause	Ukraine	perimenopausal	109	12 wks	Err 731	5	6
Heyerick, A., et al.	2006	A first prospective, randomized, double-blind, placebo-controlled study on the use of a standardized hop extract to alleviate menopausal discomforts	Maturitas	Belgium,	postmenopausal	76	12 wks	dietary supplements	4	2
Hidalgo, L. A.	2005	The effect of red clover isoflavones on menopausal symptoms, lipids and vaginal cytology in menopausal women: A randomized, double-blind, placebo-controlled study	Gynecological Endocrinology	Ecuador	postmenopausal	53	180 d	isoflavone	5	2
Hilditch, J. R.	1996	A comparison of the effects of oral conjugated equine estrogen and transdermal estradiol-17 beta combined with an oral progestin on	Maturitas	Canada	postmenopausal	84	14 wks	oral HRT	3	2

		quality of life in postmenopausal women								
Hitchcock, C. and J. Prior	2012	Oral micronized progesterone for vasomotor symptoms--a placebo-controlled randomized trial in healthy postmenopausal women.	Menapause	Canada	postmenopausal	133	16 wks	oral HRT	1	4
Holst, T. and B. Salbach	2000	Efficacy and tolerability of a new 7-day transdermal estradiol patch versus placebo in hysterectomized women with postmenopausal complaints	Maturitas	Germany	postmenopausal and surgical	221	3 mos	transdermal HRT	3	2
Honjo, H.	2009	Low-dose estradiol for climacteric symptoms in Japanese women: a randomized, controlled trial	Climacteric	Japan	postmenopausal and surgical	211	8 wks	oral HRT	4	6
Huang, A.	2015	Device-guided slow-paced respiration for menopausal HF: a randomized controlled trial	Obstetrics & Gynecology	USA	peri and postmenopausal and surgical	123	12 wks	respiration	1	6
Hudita, D.	2003	Efficacy and safety of oral tibolone 1.25 or 2.5 mg/day vs. placebo in postmenopausal women	European Review for Medical & Pharmacological Sciences	Romania	postmenopausal	162	12 wks	tibolone	3	6
Jacobson, J. S.	2001	Randomized trial of black cohosh for the treatment of HF among women with a history of breast cancer	Journal of Clinical Oncology	USA	breast cancer	85	2 mos	dietary supplements	3	2
Jenabi, E.,	2018	The effect of Valerian on the severity and	Women & Health	Iran	postmenopausal	64	2 mo	dietary supplements	5	2

		frequency of HF: A triple-blind randomized clinical trial								
Jenks, B. H.	2012	A pilot study on the effects of S-equol compared to soy isoflavones on menopausal HF frequency	Journal of Women's Health	USA	postmenopausal	102	8 wks	S-equol	5	6
Joffe, H., et al.	2014	Low-dose estradiol and the serotonin-norepinephrine reuptake inhibitor venlafaxine for vasomotor symptoms: a randomized clinical trial.	JAMA Internal Medicine	USA	peri and postmenopausal	339	8 wks	SSRI	5	6
Jou, H.	2008	Effect of intestinal production of equol on menopausal symptoms in women treated with soy isoflavones	International Journal of Gynaecology & Obstetrics	China, Taiwan	postmenopausal	96	6 mos	isoflavone	5	2
Kalay, A. E	2007	Efficacy of citalopram on climacteric symptoms	Menopause	Turkey	postmenopausal and surgical	100	8 wks	SSRI	2	2
Kaszkin-Bettag	2009	Confirmation of the efficacy of ERr 731 in perimenopausal women with menopausal symptoms	Alternative Therapies in Health & Medicine	Ukarine	perimenopausal	112	12 wks	Err 731	5	6
Khaodhiar, L.	2008	Daidzein-rich isoflavone aglycones are potentially effective in reducing HF in menopausal women	Menopause	USA	postmenopausal	147	12 wks	isoflavone	1	2
Kim, D. I.	2011	Acupuncture for HF in perimenopausal and postmenopausal women: a randomised, sham-controlled trial	Acupuncture in Medicine	South Korea	peri and postmenopausal	55	15 wks	acupuncture	3	6

Kim, K. H.	2010	Effects of acupuncture on HF in perimenopausal and postmenopausal women--a multicenter randomized clinical trial	Menopause	South Korea	peri and postmenopausal and surgical	175	8 wks	acupuncture	3	6
Kimmick, G	2006	Randomized, double-blind, placebo-controlled, crossover study of sertraline (Zoloft) for the treatment of HF in women with early stage breast cancer taking tamoxifen	Breast Journal	USA	breast cancer	62	12 wks	SSRI	3	2
Kitanohara, M., et al	2017	Effect of porcine placental extract on the mild menopausal symptoms of climacteric women	Climacteric	Japan	postmenopausal	50	12 wks	dietary supplements	5	4
Kroiss, R.	2005	The effect of tibolone in postmenopausal women receiving tamoxifen after surgery for breast cancer: a randomised, double-blind, placebo-controlled trial	BJOG	UK	breast cancer	70	12 mos	tibolone	4	6
LaCroix, A., et al.	2012	Effects of escitalopram on menopause-specific quality of life and pain in healthy menopausal women with HF: a randomized controlled trial.	Maturitas	USA,Boston, Indianapolis ,Oakland,Philadelphia	peri and postmenopausal	205	8 wks	SSRI	1	4
Lambert, M., et al.	2017	Combined Red Clover isoflavones and probiotics potently reduce menopausal vasomotor symptoms	PLoS ONE [Electronic Resource]	Denmark	perimenopausal	61	12 mos	isoflavone	4	6

Landgren, M. B	2002	Dose-response analysis of effects of tibolone on climacteric symptoms	BJOG	Sweden, the Netherlands , Finland .and Norway	postmenopausal	775	12 wks	tibolone	5	6
Lee, B. S.	2007	Efficacy and tolerability of estradiol 1 mg and drospirenone 2 mg in postmenopausal Korean women: a double-blind, randomized, placebo-controlled, multicenter study	Maturitas	South Korea	postmenopausal	158	12 wks	oral HRT	3	6
Lesi, G., et al.	2016	Acupuncture As an Integrative Approach for the Treatment of HF in Women With Breast Cancer: a Prospective Multicenter Randomized Controlled Trial (AcCliMaT).	Journal of Clinical Oncology	Italy	breast cancer	105	12 wks	acupuncture	3	6
Lewis, J. E.	2006	A randomized controlled trial of the effect of dietary soy and flaxseed muffins on quality of life and HF during menopause	Menopause	Canada	postmenopausal	99	16 wks	isoflavone	5	6
Lin, S., et al.	2011	Estradiol 1 mg and drospirenone 2 mg as hormone replacement therapy in postmenopausal Chinese women	Climacteric	China	peri and postmenopausal and surgical	249	16 wks	oral HRT	5	6
Lindh-Astrand, L.	2010	A randomized controlled study of taper-down or abrupt discontinuation of hormone therapy in women treated for vasomotor symptoms	Menopause	Sweden	postmenopausal	81	12 mos	oral HRT	2	6

Lindh-Astrand, L. and E. Nedstrand	2012	Effects of applied relaxation on vasomotor symptoms in postmenopausal women: A randomized controlled trial.	Menopause	Sweden	postmenopausal	60	6 mos	relaxation	3	6
Lipovac, M., et al.	2012	The effect of red clover isoflavone supplementation over vasomotor and menopausal symptoms in postmenopausal women.	Gynecological Endocrinology	Austria	postmenopausal	109	187d	isoflavone	3	2
Liu, J. H., et al.	2012	Synthetic conjugated estrogens-B and postmenopausal nocturnal vasomotor symptoms: a randomized controlled trial.	Obstetrics & Gynecology	USA	postmenopausal and surgical	157	12 wks	oral HRT	5	6
Liu, Zhao-min	2014	Randomized controlled trial of whole soy and isoflavone daidzein on menopausal symptoms in equol-producing Chinese postmenopausal women	Menopause	China, Hongkong	postmenopausal	270	6 mos	isoflavone	5	2
Loibl, S.	2007	Venlafaxine is superior to clonidine as treatment of HF in breast cancer patients - A double-blind, randomized study	Annals of Oncology	Germany	breast cancer	80	4 wks	SSRI	3	6
Lopes, P., et al.	2000	Randomized comparison of intranasal and transdermal estradiol	Obstetrics and Gynecology	Spain, France, Poland, Netherland, Belgium, Italy,	postmenopausal	361	16 wks	intranasal HRT	3	4

Loprinzi, C. L.	2010	Phase III, randomized, double-blind, placebo-controlled evaluation of pregabalin for alleviating HF, N07C1.[Erratum appears in J Clin Oncol. 2010 Apr 1;28(10):1808 Note: Baclueva, Ernie P [corrected to Balcueva, Ernie P]]	Journal of Clinical Oncology	USA	breast cancer	163	6 wks	pregabalin	5	6
Loprinzi, C. L.,	2000	Venlafaxine in management of HF in survivors of breast cancer: A randomised controlled trial	Lancet	USA	breast cancer	229	4 wks	SSRI	5	6
Loprinzi, C., et al.	2006	Phase III comparison of depomedroxyprogesterone acetate to venlafaxine for managing HF: north Central Cancer Treatment Group Trial N99C7	Journal of Clinical Oncology	USA	breast cancer	227	6 wks	SSRI	2	4
Luoto, R.	2012	Effect of aerobic training on HF and quality of life--a randomized controlled trial	Annals of Medicine	Finland	postmenopausal	176	24 wks	exercise	3	6
MacGregor, C. A.	2005	A randomised double-blind controlled trial of oral soy supplements versus placebo for treatment of menopausal symptoms in patients with early breast cancer	European Journal of Cancer	UK	breast cancer	72	12 wks	isoflavone	4	6
Malik, S., et a	2016	"Comparison of the symptomatic response in Indian menopausal women with different	Archives of Gynecology & Obstetrics	Indi,VMMC and SJH, New Delhia	postmenopausal and surgical	200	24 wks	oral HRT	2	6

		estrogen preparations for the treatment of menopausal symptoms: a randomized controlled trial								
Mann, E.	2012	Cognitive behavioural treatment for women who have menopausal symptoms after breast cancer treatment (MENOS 1): a randomised controlled trial	Lancet	UK	breast cancer	96	9 wks	Cognitive behavioural treatment	5	6
Mattsson, L. A.	2000	Clinical equivalence of intranasal and oral 17beta-estradiol for postmenopausal symptoms	American Journal of Obstetrics & Gynecology	Sweden, Denmark ,Spain, France, UK, Netherlands , Germany	postmenopausal	649	24 wks	intranasal HRT	5	6
Mattsson, L. A., et al.	2007	Efficacy and tolerability of continuous combined hormone replacement therapy in early postmenopausal women	Menopause International	Czech, Denmark, Finland, Germany, Hungary, Poland, Russia, Sweden	postmenopausal and surgical	459	52 wks	oral HRT	2	6
Meng, F., et al.	2017	Effect of Gua sha therapy on perimenopausal syndrome: a randomized controlled trial.	Menopause	China, Nanjing	perimenopausal	75	8 wks	GuaSha	3	2
Meuwissen, J. H.	2001	A 1-year comparison of the efficacy and clinical tolerance in postmenopausal women of two hormone replacement therapies containing estradiol in	Gynecological Endocrinology	Netherlands , Finland, Argentina, France, South Africa	postmenopausal	634	12 wks	oral HRT	3	6

		combination with either norgestrel or trimegestone								
Mizunuma, H.	2011	Clinical usefulness of a low-dose maintenance therapy with transdermal estradiol gel in Japanese women with estrogen deficiency symptoms	Climacteric	Japan	postmenopausal and surgical	209	12 wks	transdermal HRT	4	6
Mohammad-Alizadeh-Charandabi, S	2013	Efficacy of black cohosh (Cimicifuga racemosa L.) in treating early symptoms of menopause: a randomized clinical trial	Chinesische Medizin	Iran	postmenopausal	84	8 wks	dietary supplements	5	6
Morais-Socorro, M.	2012	Safety and efficacy of tibolone and menopausal transition: a randomized, double-blind placebo-controlled trial	Gynecological Endocrinology	Brazil	peri and postmenopausal	65	12 wks	tibolone	3	1
Newton, K. M., et al	2014	Efficacy of yoga for vasomotor symptoms: a randomized controlled trial.	Menopause	USA, Indianapolis ,Oakland,Ph iladelphia,	peri and postmenopausal	249	12 wks	exercise	3	6
Newton, K., et al.	2006	Treatment of vasomotor symptoms of menopause with black cohosh, multibotanicals, soy, hormone therapy, or placebo: a randomized trial.	Annals of Internal Medicine	USA, Washinton	peri and postmenopausal	351	12 mos	dietary supplements	5	4
Notelovitz, M. and J. Mattox	2000	Suppression of vasomotor and vulvovaginal symptoms with continuous oral 17beta-estradiol	Menopause	USA	postmenopausal	145	12 mos	oral HRT	2	6

Notelovitz, M., et al	2000	Initial 17beta-estradiol dose for treating vasomotor symptoms	Obstetrics and Gynecology	USA	postmenopausal	333	12 wks	oral HRT	4	2
Oktem, M.	2007	Black cohosh and fluoxetine in the treatment of postmenopausal symptoms: a prospective, randomized trial	Advances in Therapy	Turkey	postmenopausal	120	6 mos	dietary supplements	1	4
Osmers, R	2005	Efficacy and safety of isopropanolic black cohosh extract for climacteric symptoms	Obstetrics & Gynecology	Germany	postmenopausal	304	12 wks	dietary supplements	5	6
Palacios, S	2017	Omega-3 versus isoflavones in the control of vasomotor symptoms in postmenopausal women	Gynecological Endocrinology	Spain	postmenopausal	65	4 mos	isoflavone	2	6
Palacios, S.,	2004	Raloxifene is not associated with biologically relevant changes in HF in postmenopausal women for whom therapy is appropriate	American Journal of Obstetrics & Gynecology	Brazil	postmenopausal and surgical	487	8 mos	SSRI	4	4
Panay, N., et al.	2007	Ultra-low-dose estradiol and norethisterone acetate: effective menopausal symptom relief.	Climacteric	UK, USA, Finland, Switzerland	postmenopausal	577	24 wks	oral HRT	2	6
Pandya, K. J.	2005	Gabapentin for HF in 420 women with breast cancer: a randomised double-blind placebo-controlled trial	Lancet	USA	breast cancer	371	8 wks	Antiepileptics	5	4
Park, H.	2015	North Central Cancer Treatment Group N10C2 (Alliance): a double-blind placebo-	Menopause	South Korea	breast cancer	289	8 wks	Antiepileptics	3	6

		controlled study of magnesium supplements to reduce menopausal HF								
Parsey, K.	2000	Randomised, controlled comparison of transdermal estradiol with oral conjugated estrogens for the relief of HF	Clinical Drug Investigation	USA	postmenopausal	193	8 wks	oral HRT	5	4
Pinkerton, J. V.	2014	Phase 3 randomized controlled study of gastroretentive gabapentin for the treatment of moderate-to-severe HF in menopause	Menopause	USA	postmenopausal	600	12 wks	Antiepileptics	5	6
Pinkerton, J., et al.	2009	Relief of vasomotor symptoms with the tissue-selective estrogen complex containing bazedoxifene/conjugated estrogens: a randomized, controlled trial.	Menopause	USA	postmenopausal	332	12 wks	oral HRT	4	6
Pinkerton, J., et al.	2013	Desvenlafaxine compared with placebo for treatment of menopausal vasomotor symptoms: a 12-week, multicenter, parallel-group, randomized, double-blind, placebo-controlled efficacy trial	Menopause	USA	postmenopausal	365	12 wks	SSRI	4	4
Plotnikoff, G. A.	2011	The TU-025 keishibukuryogan clinical trial for HF management in postmenopausal women: results and	Menopause	USA	postmenopausal and surgical	178	12 wks	keishibukuryogan	3	4

		lessons for future research								
Pockaj, B. A.	2006	Phase III double-blind, randomized, placebo-controlled crossover trial of black cohosh in the management of HF: NCCTG Trial N01CC1	Journal of Clinical Oncology	USA	breast cancer	132	8 wks	dietary supplements	3	4
Polisseni, A. F.,	2013	Effects of a continuous-combined regimen of low-dose hormone therapy (oestradiol and norethindrone acetate) and tibolone on the quality of life in symptomatic postmenopausal women: a double-blind, randomised study	Maturitas	Brazil	postmenopausal	174	12 wks	tibolone	5	6
Pornel, B.	1996	Efficacy and safety of Menorest in two positive-controlled studies	European Journal of Obstetrics, Gynecology, & Reproductive Biology	UK, Italy, Newzealand , Australia, Belgium, Netherland, France	postmenopausal	214	12 wks	oral HRT	2	2
Pornel, B.	2005	A study of the control of climacteric symptoms in postmenopausal women following sequential regimens of 1 mg 17beta-estradiol and trimegestone compared with a regimen containing 1 mg estradiol valerate and norethisterone over a two-year period	Gynecological Endocrinology	Belgium, Czech, France,Germany, Switzerland, Isarel, UK, Netherlands	postmenopausal	1218	cycle 13	oral HRT	4	6
Pornel, B., et al	1995	Efficacy and tolerability of Menorest 50 compared with	Maturitas	Belgium, Finland,	postmenopausal	205	12 wks	oral HRT	2	6

		Estraderm TTS 50 in the treatment of postmenopausal symptoms. A randomized, multicenter, parallel group study		Sweden, Netherlands						
Prague, J. K	2017	Neurokinin 3 receptor antagonism as a novel treatment for menopausal HF: a phase 2, randomised, double-blind, placebo-controlled trial	Lancet	UK	postmenopausal	68	4 wks	Neurokinin 3	5	6
Pruthi, S.	2012	A phase III, randomized, placebo-controlled, double-blind trial of flaxseed for the treatment of HF: North Central Cancer Treatment Group N08C7	Menopause	USA	breast cancer	188	6 wks	dietary supplements	4	6
Raynaud, J. P.	2005	Comparison of the efficacy and tolerability of a new once-a-week matricial estradiol transdermal system (Estrapatch 40 and Estrapatch 60) with a twice week system	Journal of Steroid Biochemistry & Molecular Biology	Austria	postmenopausal	496	4 wks	transdermal HRT	2	6
Reddy, S. Y.	2006	Gabapentin, estrogen, and placebo for treating HF: a randomized controlled trial	Obstetrics & Gynecology	USA	postmenopausal and surgical	60	12 wks	Antiepileptics	5	4
Rovati, L. C.,	2000	Dose-response efficacy of a new estradiol transdermal matrix patch for 7-day application: a randomized, double-	Gynecological Endocrinology	Italy	postmenopausal and surgical	311	12 wks	transdermal HRT	4	6

		blind, placebo-controlled study. Italian Menopause Research Group								
Rozenbaum, H., et al.	2002	Efficacy and tolerability of pulsed estrogen therapy: A 12-week double-blind placebo-controlled study in highly symptomatic postmenopausal women.	Climacteric	France	postmenopausal	165	8 wks	intranasal HRT	3	4
Rozenberg, S.	1997	Comparison of continuous and sequential transdermal progestogen with sequential oral progestogen in postmenopausal women using continuous transdermal estrogen: vasomotor symptoms, bleeding patterns, and serum lipids	International Journal of Fertility & Womens Medicine	Belgium Finland, Sweden, UK, Netherlands	postmenopausal	774	14 wks	transdermal HRT	2	3
Saensak, S.	2013	Effectiveness of a modified version of the applied relaxation technique in treatment of perimenopausal and postmenopausal symptoms	International Journal of Women's Health	Thailand	peri and postmenopausal and surgical	105	12 wks	relaxation	3	2
Sangwan, P., et al.	2015	A comparative study of efficacy and safety of red clover versus conjugated estrogen on vasomotor symptoms and sleep patterns in postmenopausal women.	Indo Global Journal of Pharmaceutical Sciences	India	postmenopausal	50	12 wks	dietary supplements	2	4

Schellenberg, R., et al.	2012	Dose-dependent effects of the Cimicifuga racemosa extract Ze 450 in the treatment of climacteric complaints: A randomized, placebo-controlled study	Evidence-based Complementary and Alternative Medicine	Switzland	postmenopausal	105	12 wks	dietary supplements	3	6
Schurmann, R.	2004	Estradiol and drospirenone for climacteric symptoms in postmenopausal women: a double-blind, randomized, placebo-controlled study of the safety and efficacy of three dose regimens	Climacteric	Germany, Netherland	postmenopausal	225	16 wks	oral HRT	4	6
Secreto, G., et al.	2004	Soy isoflavones and melatonin for the relief of climacteric symptoms: a multicenter, double-blind, randomized study.	Maturitas	Italy	postmenopausal	262	4 mos	isoflavone	5	6
Sehhatie Shafaie, F.	2014	Effect of Education through Support -Group on Early Symptoms of Menopause: a Randomized Controlled Trial	Journal of Caring Sciences	Iran	postmenopausal	124	4 wks	education	3	6
Shamshad Begum, S.	2016	A Novel Extract of Fenugreek Husk (FenuSMARTTM) Alleviates Postmenopausal Symptoms and Helps to Establish the Hormonal Balance: A Randomized, Double-	Phytotherapy Research	India	postmenopausal	88	90 d	dietary supplements	4	2

		Blind, Placebo-Controlled Study							
Shobeiri, F.	2017	The Effect of Educational Program on Quality of Life in Menopausal Women: A Clinical Trial	Journal of menopausal medicine	Iran	postmenopausal	100	3 mos	education	2	1
Shulman, L. P.,	2002	Safety and efficacy of a continuous once-a-week 17beta-estradiol/levonorgestrel transdermal system and its effects on vasomotor symptoms and endometrial safety in postmenopausal women: the results of two multicenter, double-blind, randomized, controlled trials	Menopause	USA	peri and postmenopausal	293	4 wks	transdermal HRT	4	6
Simon, J	2006	Estradiol in micellar nanoparticles: the efficacy and safety of a novel transdermal drug-delivery technology in the management of moderate to severe vasomotor symptoms	Menopause	USA	postmenopausal and surgical	200	12 wks	transdermal HRT	4	6
Simon, J. A	2013	Low-dose paroxetine 7.5 mg for menopausal vasomotor symptoms: two randomized controlled trials	Menopause	USA	postmenopausal and surgical	591	24 wks	SSRI	5	4
Simon, J. A., et al	2001	Perimenopausal women in estrogen vasomotor trials: contribution to placebo effect and efficacy outcome.	Menopause	USA	postmenopausal and surgical	120	12 wks	oral HRT	4	4

Simon, J., et al	2016	Extended-release oxybutynin therapy for vasomotor symptoms in women: a randomized clinical trial.	Menopause	USA	postmenopausal and surgical	148	12 wks	oxybutynin	5	6
Simon, J., et al.	2007	Low dose of transdermal estradiol gel for treatment of symptomatic postmenopausal women: a randomized controlled trial.	Obstetrics & Gynecology	USA, Canada	postmenopausal and surgical	484	12 wks	transdermal HRT	5	6
Sluijs, C., et al.	2009	randomized placebo-controlled trial on the effectiveness of an herbal formula to alleviate menopausal vasomotor symptoms.	Menopause	Australia	postmenopausal	93	16 wks	dietary supplements	5	6
Sood, R., et al.	2013	Paced breathing compared with usual breathing for HF	Menopause	USA	breast cancer	93	9 wks	respiration	2	2
Speroff, L	2003	Efficacy and tolerability of a novel estradiol vaginal ring for relief of menopausal symptoms."	Obstetrics & Gynecology	USA	postmenopausal	333	13 wks	HRT vaginal ring	3	4
Speroff, L., et al	2008	Efficacy and tolerability of desvenlafaxine succinate treatment for menopausal vasomotor symptoms: a randomized controlled trial	Obstetrics & Gynecology	USA	postmenopausal	612	12 wks	SSRI	5	6
Speroff, L., et al.	2006	"Efficacy of a new, oral estradiol acetate formulation for relief of menopause symptoms.	Menopause	USA	postmenopausal and surgical	552	12 wks	oral HRT	5	6
Speroff, L., et al.	2000	The effect of varying low-dose combinations of norethindrone	Meopause	USA	postmenopausal and surgical	485	4 wks	oral HRT	2	2

		acetate and ethinyl estradiol (femhrt) on the frequency and intensity of vasomotor symptoms							
Speroff, L., et al.	1996	Efficacy and local tolerance of a low-dose, 7-day matrix estradiol transdermal system in the treatment of menopausal vasomotor symptoms.	Obstetrics & Gynecology	USA	postmenopausal and surgical	324	12 wks	transdermal HRT	4	2
St Germain, A., et al.	2001	Isoflavone-rich or isoflavone-poor soy protein does not reduce menopausal symptoms during 24 weeks of treatment	Menopause	USA	perimenopausal	69	24 wks	isoflavone	2	6
Stearns, V., et al.	2003	Paroxetine controlled release in the treatment of menopausal HF: a randomized controlled trial.	JAMA	USA	postmenopausal and surgical	165	6 wks	SSRI	4	6
Steels, E., et al.	2017	Efficacy of a Proprietary Trigonella foenum-graecum L. De-Husked Seed Extract in Reducing Menopausal Symptoms in Otherwise Healthy Women: a Double-Blind, Randomized, Placebo-Controlled Study	Phytotherapy research : PTR	Australis,	peri and postmenopausal	115	12 wks	dietary supplements	5	6
Steels, E., et al.	2018	A double-blind, randomized, placebo-controlled trial evaluating safety and efficacy of an ayurvedic	Journal of Herbal Medicine.	Australia	postmenopausal	117	12 wks	dietary supplements	5	6

		botanical formulation in reducing menopausal symptoms in otherwise healthy women."								
Sternfeld, B., et al.	2014	Efficacy of exercise for menopausal symptoms: a randomized controlled trial	Menopause	USA	peri and postmenopausal and surgical	142	12 wks	exercise	5	6
Stevenson, J. C., et al.	2010	Oral ultra-low dose continuous combined hormone replacement therapy with 0.5 mg 17beta-oestradiol and 2.5 mg dydrogesterone for the treatment of vasomotor symptoms: results from a double-blind, controlled study."	Maturitas	France, Poland ,Romania, Russia	postmenopausal	313	13 wks	oral HRT	4	6
Stovall, D., et al.	2007	The effects of combined raloxifene and oral estrogen on vasomotor symptoms and endometrial safety	Menopause	USA	postmenopausal	149	52 wks	SSRI	4	6
Studd, J.	1999	Efficacy and acceptability of intranasal 17 beta-oestradiol for menopausal symptoms: randomised dose-response study. Aerodiol Study Group	Lancet	Denmark, UK	postmenopausal	420	12 wks	intranasal HRT	5	4
Studd, J., et al.	1995	Efficacy and tolerance of Menorest compared to Premarin in the treatment of postmenopausal women. A randomised, multicentre, double-	Maturitas	UK, Italy, Australia, New Zealand	postmenopausal and surgical	214	12 wks	oral HRT	2	6

		blind, double-dummy study.								
Sulak, P. J.	1999	Efficacy and safety of a constant-estrogen, pulsed-progestin regimen in hormone replacement therapy	International Journal of Fertility & Womens Medicine	USA	postmenopausal	1253	3 mos	oral HRT	2	4
Suvanto-Luukkonen, E., et al	2005	Citalopram and fluoxetine in the treatment of postmenopausal symptoms: a prospective, randomized, 9-month, placebo-controlled, double-blind study.	Menopause	Finland	postmenopausal	150	9 mo	SSRI	4	2
Swanson, S. G., et al	2006	"Tibolone for the treatment of moderate to severe vasomotor symptoms and genital atrophy in postmenopausal women: a multicenter, randomized, double-blind, placebo-controlled study.	Menopause	USA	postmenopausal	396	12 wks	tibolone	4	6
Tanmahasamut, P.	2015	Cimicifuga racemosa extract for relieving menopausal symptoms: a randomized controlled trial	Climacteric	Thailand	peri and postmenopausal	54	12 wks	dietary supplements	5	2
Tice, J. A.	2003	Phytoestrogen supplements for the treatment of HF: the Isoflavone Clover Extract (ICE) Study: a randomized controlled trial	JAMA	USA	postmenopausal and surgical	252	12 wks	isoflavone	5	6
Uebelhack, R.	2006	Black cohosh and St. John's wort for	Obstetrics and Gynecology	Germany	postmenopausal	206	16 wks	dietary supplements	4	4

		climacteric complaints: A randomized trial							
Utian, W. H., et al.	1999	Efficacy and safety of low, standard, and high dosages of an estradiol transdermal system (Esclim) compared with placebo on vasomotor symptoms in highly symptomatic menopausal patients. The Esclim Study Group	American Journal of Obstetrics & Gynecology	USA	postmenopausal and surgical	196	12 wks	oral HRT	4	2
Utian, W., et al.	2004	Relief of HF with new plant-derived 10-component synthetic conjugated estrogens.	Obstetrics and Gynecology	USA	postmenopausal and surgical	281	12 wks	dietary supplements	4	2
Utian, W., et al.	2005	Comparative controlled trial of a novel oral estrogen therapy, estradiol acetate, for relief of menopause symptoms.	Menopause	USA	postmenopausal and surgical	249	12 wks	oral HRT	4	6
van der Sluijs, C. P., et al.	2009	A randomized placebo-controlled trial on the effectiveness of an herbal formula to alleviate menopausal vasomotor symptoms.	Menopause	Australis, Syndey	postmenopausal	93	20 wks	dietary supplements	1	6
van Die, M. D.	2009	Hypericum perforatum with Vitex agnus-castus in menopausal symptoms: a randomized, controlled trial	Menopause	Australia	peri and postmenopausal	92	12 wks	dietary supplements	5	6
Van Patten, C. L., et al.	2002	Effect of soy phytoestrogens on HF in postmenopausal women with breast cancer: a randomized, controlled clinical trial.	Journal of Clinical Oncology	Canada, British Columbia	breast cancer	157	12 wks	isoflavone	4	6

Verhoeven, M. O., et al	2005	Effect of a combination of isoflavones and Actaea racemosa Linnaeus on climacteric symptoms in healthy symptomatic perimenopausal women: a 12-week randomized, placebo-controlled, double-blind study.	Menopause	Netherlands	perimenopausal	124	12 wks	isoflavone	5	6
Villa, P.	2017	The impact of combined nutraceutical supplementation on quality of life and metabolic changes during the menopausal transition: a pilot randomized trial	Archives of Gynecology & Obstetrics	Italy	perimenopausal	90	6 mos	dietary supplements	3	4
Vincent, A.	2007	Acupuncture for HF: a randomized, sham-controlled clinical study	Menopause	USA	peri and postmenopausal	103	12 wks	Antiepileptics	2	4
von Hagens	2012	Treating menopausal symptoms with a complex remedy or placebo: a randomized controlled trial	Climacteric	Germany	postmenopausal	102	12 wks	dietary supplements	5	6
von Holst, T.	2002	Efficacy of a new 7-day transdermal sequential estradiol/levonorgestrel patch in women	Maturitas	Germany	postmenopausal and surgical	216	12 wks	transdermal HRT	4	4
Walker, E. M., et al.	2010	Acupuncture versus venlafaxine for the management of vasomotor symptoms in patients with hormone receptor-positive breast cancer: a randomized controlled trial	Journal of Clinical Oncology	USA, Michigan	breast cancer	50	12 wks	SSRI	2	3

Wang, C. C.	2013	A randomized, double-blind, multiple-dose escalation study of a Chinese herbal medicine preparation (Dang Gui Buxue Tang) for moderate to severe menopausal symptoms and quality of life in postmenopausal women	Menopause	China	postmenopausal	60	12 wks	dietary supplements	5	4
Winther, K	2005	Femal, a herbal remedy made from pollen extracts, reduces HF and improves quality of life in menopausal women: a randomized, placebo-controlled, parallel study	Climacteric	Sweden	postmenopausal	50	4 wks	dietary supplements	5	6
Wong, C.	2018	Mindfulness-Based Stress Reduction (MBSR) or Psychoeducation for the Reduction of Menopausal Symptoms: A Randomized, Controlled Clinical Trial	Scientific Reports	China, Hongkong	peri and postmenopausal	197	8 mos	mindfulness training	3	6
Wuttke, W	2006	Efficacy and tolerability of the Black cohosh (Actaea racemosa) ethanolic extract BNO 1055 on climacteric complaints: A double-blind, placebo- and conjugated estrogens-controlled study	Maturitas	Czech	postmenopausal	95	12 wks	dietary supplements	5	4
Wyrwich, K. W., et al	2008	Identifying meaningful differences in vasomotor symptoms	Menopause	US	postmenopausal and surgical	620	12 wks	SSRI	5	6

		among menopausal women								
Xi, S.	2017	Effect of health education combining diet and exercise supervision in Chinese women with perimenopausal symptoms: a randomized controlled trial	Climacteric	China	perimenopausal	60	12 wks	education	3	2
Xia, Y., et al.	2012	A randomized double-blind placebo-controlled trial of a Chinese herbal medicine preparation (Jiawei Qing'e Fang) for HF and quality of life in perimenopausal women	Menopause	China, Tianjin	perimenopausal	72	12 wks	dietary supplements	5	6
Yang, H.,	2012	Effect of combining therapy with traditional chinese medicine-based psychotherapy and herbal medicines in women with menopausal syndrome: a randomized controlled clinical trial	Evidence-Based Complementary & Alternative Medicine: eCAM	China	postmenopausal	424	12 wks	dietary supplements	5	6
Zhong, L. L.	2013	A randomized, double-blind, controlled trial of a Chinese herbal formula (Er-Xian decoction) for menopausal symptoms in Hong Kong perimenopausal women	Menopause	China, Hongkong	peri and postmenopausal	108	12 wks	dietary supplements	5	6
