# Supplementary material for: Variation in menopausal vasomotor symptoms outcomes in clinical trials: a systematic review
Source: BJOG. 2019 Nov 13;127(3):320–33. doi: 10.1111/1471-0528.15990 (PMC6972542; doi:10.1111/1471-0528.15990)
Supplement: Supplementary file 2 — Table S2. Vasomotor‐related outcomes measured by diary. [file BJO-127-320-s002.pdf]

Table S2. Vasomotor related outcomes measured by diary

Diary measures (as defined in each paper)	n
Frequency of HF	28
Frequency of HF/NS	19
Frequency of moderate to severe HF	7
Frequency of moderate to severe HF/NS	4
Number of HF	28
Number of HF/NS	6
Number of moderate to severe HF	9
Number of moderate to severe HF/NS	10
Severity of HF	13
Severity of HF/NS	10
Severity of moderate to severe HF	5
Severity of moderate to severe HF/NS	6
Intensity of HF	5
Intensity of HF/NS	2
Incidence of HF	1
Bothersomeness of HF/NS	5
HF (composite/severity) score	34
A 40%-75% reduction in HF	8
Frequency of awakenings resulting from nocturnal vasomotor symptoms	1
More than 50% patients halved the distress from HF/NS	1
Moderate to severe rate of HF	1
Percentage of HF reported	1
Proportion of women responding to treatment	1
Number of Vasomotor complaints	1
The percentage change in HF score	1

* Expression of the primary outcomes as quoted in each study

Abbreviations: Hot flushes (HF), night sweats (NS)
