# Supplementary material for: Variation in menopausal vasomotor symptoms outcomes in clinical trials: a systematic review
Source: BJOG. 2019 Nov 13;127(3):320–33. doi: 10.1111/1471-0528.15990 (PMC6972542; doi:10.1111/1471-0528.15990)
Supplement: Supplementary file 3 — Table S3. Different definitions for frequency, severity, intensity, and vasomotor scores of vasomotor‐related outcomes. [file BJO-127-320-s003.pdf]

Table S3. Different definitions for frequency, severity, intensity and vasomotor scores of vasomotor related outcomes

Definitions	n
**Frequency**	**97**
Defined as number	76
Numeric visual scale from 0-10	2
Occurrence of HF	1
Not defined	18
**Severity**	**111**
4-point scale	70
5-point scale	21
7-point Likert scale	1
Visual analog scale	5
Defined as transient episodes of HF	1
Defined as four levels of HF severity from four domains	1
Defined as percentage of number of HF/NS decreased	1
Severity "bubble" on a scale of 1 to 7	1
Defined as adding the duration and intensity score	1
Not defined	9
**Intensity**	**28**
Same as 4-point severity scale	15
Same as 5-point severity scale	5
Five-step self-rating scale	1
Defined as cumulative area under the curve of each HF	1
Defined as 5-point scale bothersomeness of HF	1
Total number of moderate or severe HF	1
Visual analog scale	1
Not defined	3
**HF composite/severity/intensity score**	**68**
HF severity score	27
Moderate to severe HF severity score	1
Average of HF severity score	14
Average of moderate to severe HF severity score	6
Daily average of HF severity score	4
Sum of HF severity score	1
Total number of HF multiplied by average severity	6
Sum of all level of severity	2
Sum of the duration and intensity score	1
5-point scale=severity	1
4-point verbal scale	1
Not clear	3
Not defined	1

- HF severity score=number of HF weighted by severity
- Moderate to severe HF severity score= number of moderate to severe HF weighted by severity
- Average of HF severity score=HF severity score divided by total number of HF
- Average of moderate to severe HF severity score= Moderate to severe HF severity score divided by total number of HF
- Average day of HF severity score=HF severity score divided by sum of day
- Sum of HF severity score=total number of HF weighted by severity

Abbreviations: Hot flushes (HF), night sweats (NS)
