# Supplementary material for: Variation in menopausal vasomotor symptoms outcomes in clinical trials: a systematic review
Source: BJOG. 2019 Nov 13;127(3):320–33. doi: 10.1111/1471-0528.15990 (PMC6972542; doi:10.1111/1471-0528.15990)
Supplement: Supplementary file 4 — Appendix S1. Search strategy. [file BJO-127-320-s004.pdf]

Appendix S1. Search Strategy

Cochrane Central Register of Controlled Trials

#1 MeSH descriptor: [Menopause] explode all trees（6786）

#2 MeSH descriptor: [Climacteric] explode all trees（7012）

#3 (menopaus* or perimenopaus* or postmenopaus* or climacter*):ti,ab（16690）

#4 #1 or #2 or #3 （17709）

#5 MeSH descriptor: [Hot Flashes] explode all trees（706）

#6 (fl?sh* or hot fl*sh* or sweat* or night sweat* or sleep hyperhidrosis or nocturnal diaphoresis):ti,ab（5415）

#7 #5 or #6 （5588）

#8 vasomotor:ti,ab（1327）

#9 #4 and #7 and #8（430）

Embase 1996 to 2018 Week 14

1. exp menopause/ or exp menopause related disorder/ or exp "menopause and climacterium"/ （105520）
2. exp climacterium/（5967）
3. (menopaus* or perimenopaus* or postmenopaus* or climacter*).tw.（99849）
4. 1 or 2 or 3（129295）
5. exp hot flush/（13499）
6. (fl?sh* or hot fl*sh* or sweat* or night sweat* or sleep hyperhidrosis or nocturnal diaphoresis).tw. （69294）
7. 5 or 6（77598）
8. vasomotor.tw.（9975）
9. 4 and 7 and 8（1383）

Ovid MEDLINE(R) In-Process & Other Non-Indexed Citations and Ovid MEDLINE(R) 1946 to Present

1. exp Menopause/（52828）
2. exp CLIMACTERIC/（55466）
3. (menopaus* or perimenopaus* or postmenopaus* or climacter*).tw（83563）
4. 1 or 2 or 3（97576）
5. exp Hot Flashes/（2892）
6. (fl?sh* or hot fl*sh* or sweat* or night sweat* or sleep hyperhidrosis or nocturnal diaphoresis).tw. （67556）
7. 5 or 6（68552）
8. vasomotor.tw.（11905）
9. 4 and 7 and 8（1121）
